# Organ-Specific Mitochondrial DNA Copy Number Responses in Neonatal Mice Following in Utero Irradiation

**DOI:** 10.3390/ijms27177671

**Published:** 2026-08-27

**Authors:** Haruka Kubo, Ryosuke Seino, Hisanori Fukunaga

**Affiliations:** 1 Division of Health Sciences, Graduate School of Health Sciences, Hokkaido University, Sapporo 060-0812, Japan; kubo.haruka.c9@elms.hokudai.ac.jp; 2 Department of Radiological Technology, Faculty of Health Sciences, Hokkaido University of Science, Sapporo 006-8585, Japan; seino-r@hus.ac.jp; 3 Department of Biomedical Science and Engineering, Faculty of Health Sciences, Hokkaido University, Sapporo 060-0812, Japan; 4 Center for Environmental and Health Sciences, Hokkaido University, Sapporo 060-0812, Japan

**Keywords:** developmental origins of health and disease, in utero irradiation, mitochondrial DNA, PCR, X-ray

## Abstract

Early-life ionizing radiation exposure may induce molecular responses without changes in birth outcomes. We examined whether in utero exposure to 0.2 Gy X-rays affects mitochondrial DNA copy number (mtDNAcn) and an index estimating the proportion of mitochondrial DNA (mtDNA) molecules amplifiable across a defined long region. Pregnant mice received sham irradiation or exposure on embryonic day 8 (E8) or 15 (E15). Offspring brain, heart, and liver were examined at birth, with litter means used as experimental units. Litter size and neonatal body weight showed no group differences. In organ-wise comparisons, brain mtDNAcn was higher after E8 and E15 exposure, heart mtDNAcn was higher after E15 exposure, and liver mtDNAcn showed no statistically detectable difference. Mixed-effects analysis supported an organ-dependent pattern of mtDNAcn responses, although direct E8-versus-E15 comparisons were not significant within any organ. The estimated proportion of long-fragment-amplifiable mtDNA was lower in the brain after E15 exposure in the organ-wise analysis, but mixed-effects analysis did not support organ- or gestational-stage-dependent patterns in this measure. Thus, in utero X-ray exposure produced organ-dependent mtDNAcn responses at birth but no demonstrable gestational-stage-specific response within individual organs.

## 1. Introduction

Early life is a sensitive period during which environmental stressors can induce biological alterations that are not necessarily captured by conventional birth outcomes. In developmental and environmental health research, traditional endpoints such as litter size and birth weight remain important, but they may be insufficient to detect early molecular responses that precede or occur independently of overt phenotypic changes. Identifying sensitive molecular endpoints at birth is therefore important for understanding how prenatal exposure may influence organ development and later-life vulnerability.

Ionizing radiation is a physical environmental stressor that can perturb redox homeostasis and mitochondrial function, including alterations in mitochondrial DNA (mtDNA) maintenance [1]. Because mtDNA is maintained through continuous replication and quality-control processes, mtDNA copy number (mtDNAcn) and mtDNA integrity may reflect a dynamic balance between stress response, compensatory replication, and genome maintenance. Previous studies have shown that radiation exposure can alter mtDNAcn in a dose- and time-dependent manner, highlighting the dynamic nature of mitochondrial genomic regulation in vitro and in vivo [2,3,4]. In addition to mtDNAcn, the long-fragment polymerase chain reaction (PCR)-based intact mtDNA ratio provides a complementary indicator of the amplifiability of a defined long mtDNA region by estimating the relative abundance of mtDNA molecules that remain amplifiable across that region. In vivo studies using this approach have suggested that increased mtDNAcn can accompany a reduced intact mtDNA ratio after radiation exposure, raising the possibility that mitochondrial genome quantity and the amplifiability of a defined long mtDNA region are jointly remodeled under stress [4,5].

The prenatal period includes distinct windows of organogenesis and fetal maturation [6,7]. Radiation exposure during gestation can affect the developing embryo or fetus in a timing- and dose-dependent manner, but low-dose exposure may not produce overt developmental abnormalities detectable at birth [8,9,10,11]. Recent work has further suggested that in utero X-ray exposure alters mtDNAcn in mothers and offspring even in the absence of obvious developmental effects [5]. However, how mitochondrial genomic responses to in utero exposure are organized across neonatal organs remains unclear. It therefore remains unclear whether the gestational timing of exposure differentially affects mtDNA quantity and the amplifiability of a defined long mtDNA region across neonatal organs.

Radiation exposure before conception and during gestation represents biologically distinct contexts. Preconception exposure primarily involves parental germ cells and is therefore associated with hereditary or intergenerational effects, whereas in utero exposure directly affects the developing embryo or fetus and may result in developmental, carcinogenic, and hereditary consequences depending on timing and dose [9,10,11]. These distinctions suggest that mitochondrial genomic responses may be differentially organized according to exposure timing and tissue context. Mitochondrial DNA copy number is regulated in a tissue-specific manner [12]. Moreover, mitochondrial respiratory-chain composition, oxidative capacity, and energy metabolism undergo substantial early postnatal maturation in the brain, heart, and liver, respectively [13,14,15]. These developmental differences provide a biological rationale for examining mitochondrial genomic responses separately in these organs. Whether in utero irradiation elicits organ-dependent changes in both mtDNA quantity and the amplifiability of a defined long mtDNA region remains unresolved.

**Figure 1:**
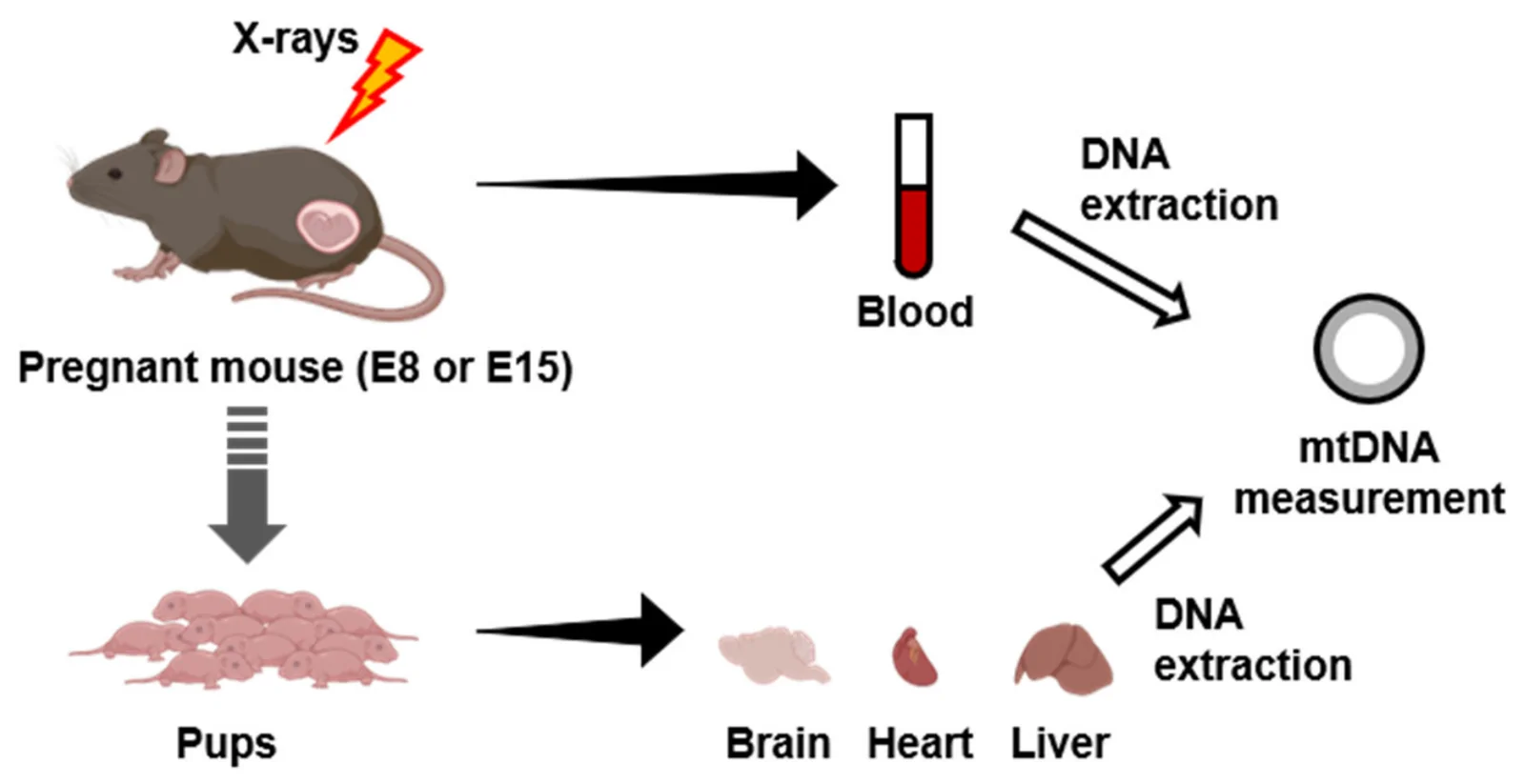
Experimental design. Pregnant C57BL/6N mice were sham-irradiated or exposed to a single whole-body dose of 0.2 Gy X-rays at E8 or E15, and offspring brain, heart, and liver tissues were collected at birth for analysis of mtDNA indices. Created in BioRender. Fukunaga, H. (2026) https://app.biorender.com/citation/6a635e567a1abfd955d3795b.

In the present study, we aimed to determine whether in utero exposure to 0.2 Gy X-rays at embryonic day 8 (E8) or embryonic day 15 (E15) affects mtDNA copy number and an mtDNAcn-normalized index derived from the amplification of a defined 8.2-kb mtDNA region in neonatal mouse organs. We measured these indices in the brain, heart, and liver at birth and evaluated whether the response patterns differed among organs and between the two exposure timings. The experimental design is shown in Figure 1.

## 2. Results

### 2.1. Maternal Parameters and Gross Birth Outcomes

**Figure 2:**
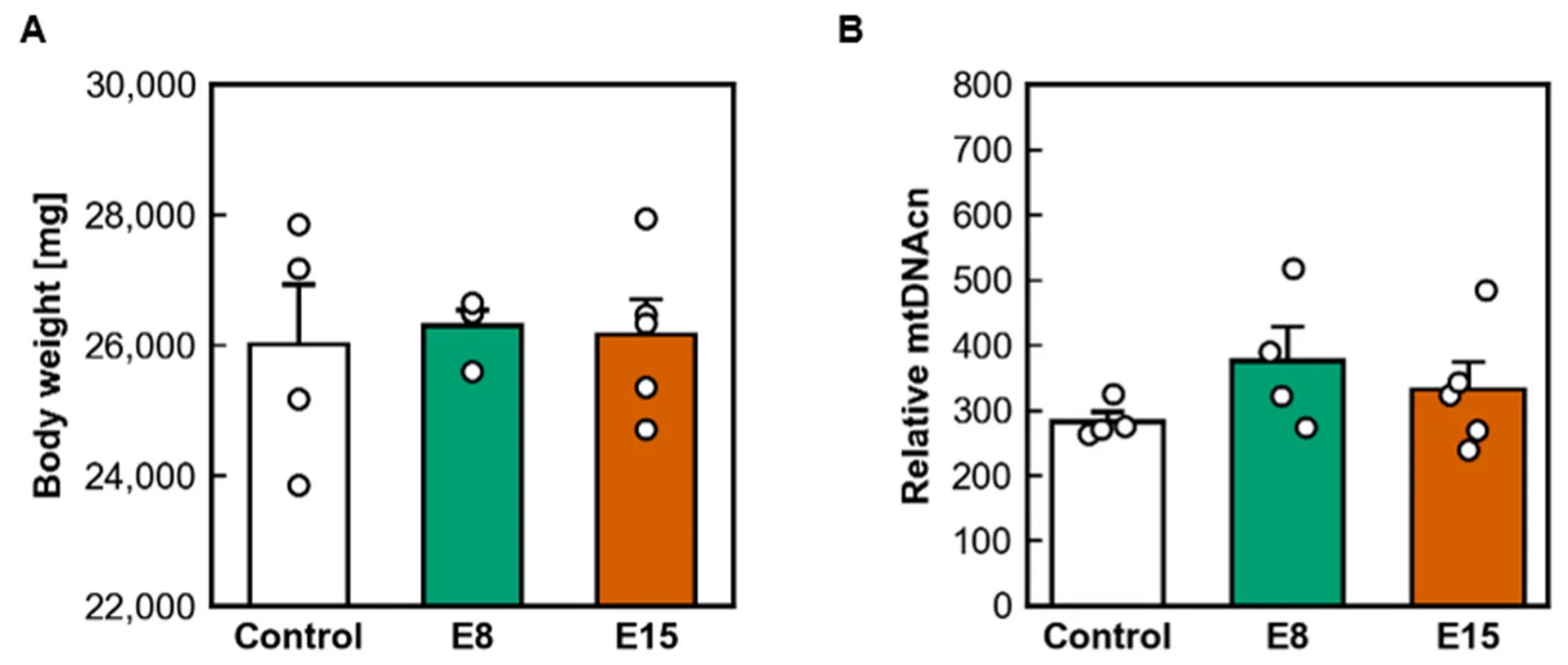
Maternal parameters at E8 before irradiation. (**A**) Maternal body weight. (**B**) Peripheral blood-derived mtDNAcn. Data are presented as mean ± SEM. Statistical analysis was performed using one-way ANOVA followed by Tukey–Kramer multiple-comparison tests.

Maternal parameters at E8 prior to irradiation are summarized in Figure 2. No statistically detectable differences in maternal body weight were observed among the groups. (control: 26,010 ± 918 mg; E8: 26,305 ± 241 mg; E15: 26,160 ± 551 mg) (Figure 2A). Similarly, no statistically detectable group difference in maternal peripheral blood-derived mtDNAcn was observed (control: 283 ± 14; E8: 375 ± 53; E15: 332 ± 43) (Figure 2B).

**Figure 3:**
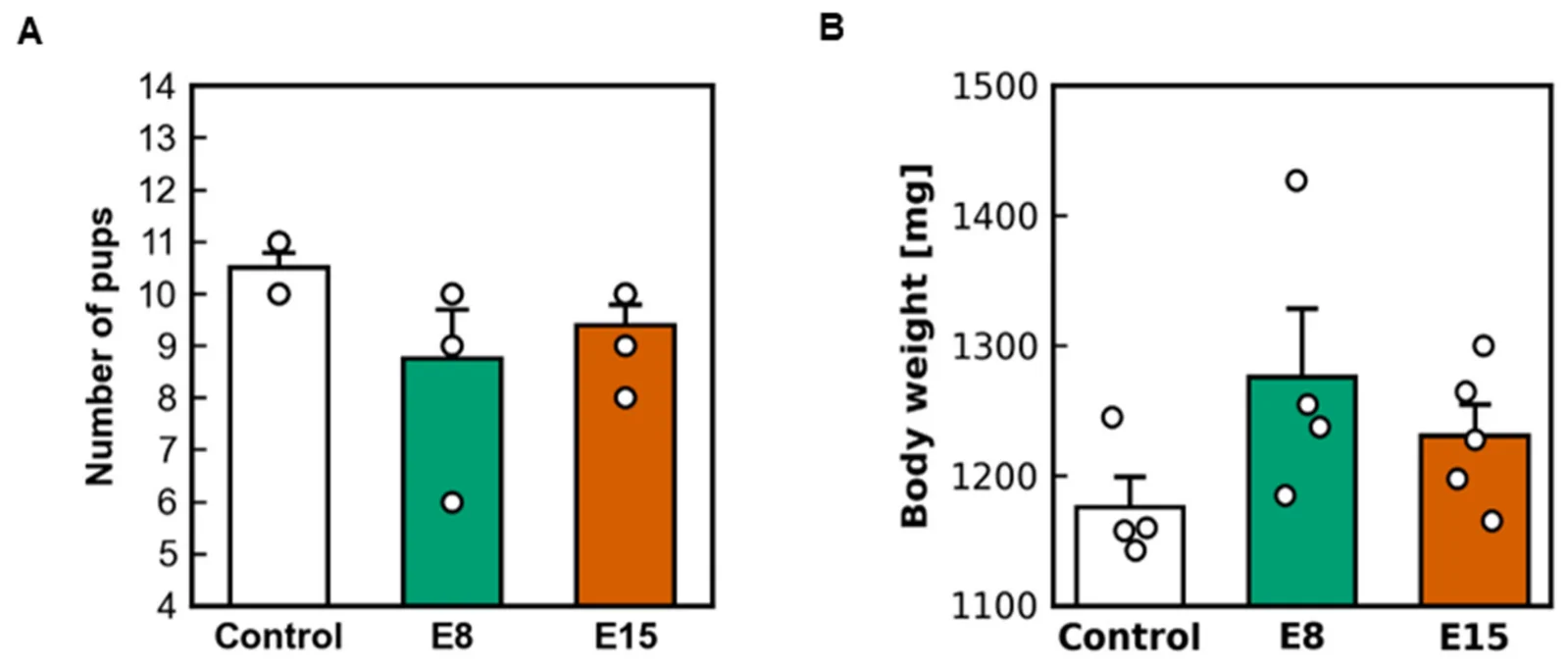
Litter outcomes at birth. (**A**) Litter size. (**B**) Neonatal body weight. Body weight was averaged among four pups within each litter. Data are presented as mean ± SEM; each point represents one litter. Statistical analysis was performed using one-way ANOVA followed by Tukey–Kramer multiple-comparison tests.

Litter outcomes are summarized in Figure 3. The number of pups per litter did not differ significantly among the control, E8-irradiated, and E15-irradiated groups (control: 10.5 ± 0.3; E8: 8.8 ± 0.9; E15: 9.4 ± 0.4) (Figure 3A). Similarly, no significant differences were observed in neonatal body weight among the groups (control: 1176.2 ± 23.2 mg; E8: 1276.2 ± 52.6 mg; E15: 1231.0 ± 23.9 mg) (Figure 3B). No statistically detectable differences in litter size or neonatal body weight were observed among the groups.

### 2.2. Mitochondrial DNA Indices in Offspring at Birth

**Figure 4:**
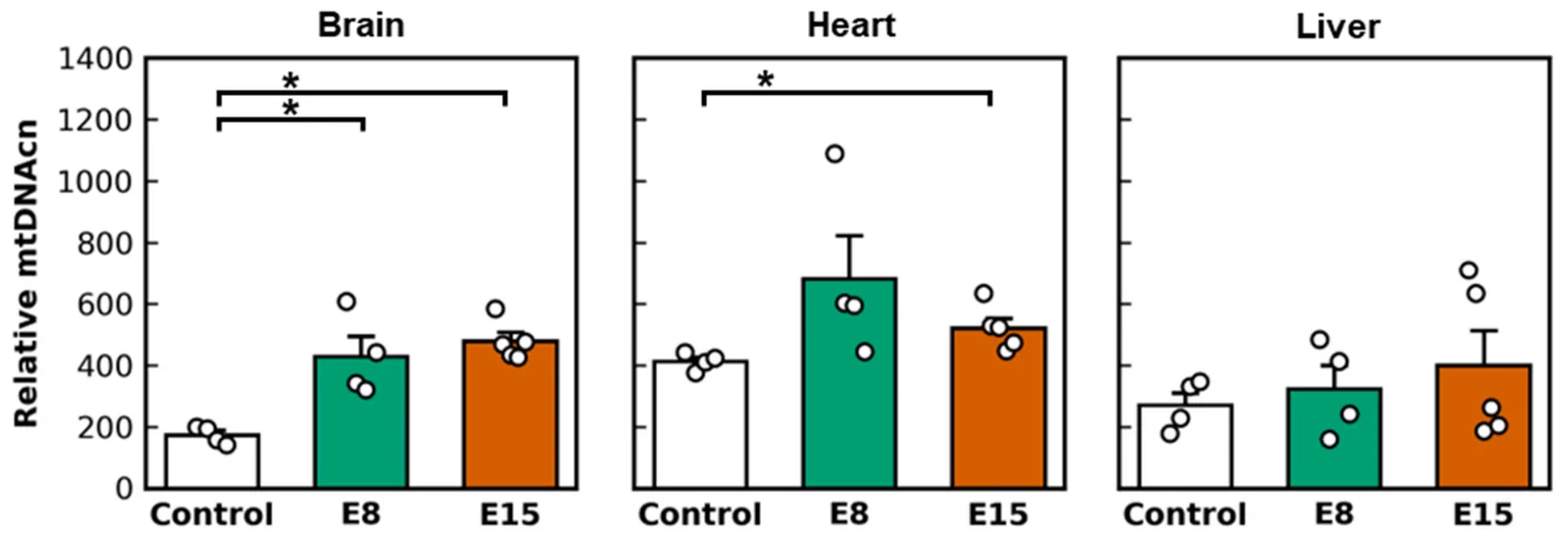
Tissue mtDNAcn in offspring at birth. Data are presented as mean ± SEM; each point represents one litter (Control, *n* = 4; E8, *n* = 4; E15, *n* = 5). Statistical analysis was performed using the Kruskal–Wallis test followed by Steel–Dwass multiple-comparison tests. * *p* < 0.05.

As shown in Figure 4, mtDNAcn showed organ-dependent responses. Brain mtDNAcn was significantly increased in both the E8 and E15 groups compared with the control group (control: 174 ± 14; E8: 428 ± 65; E15: 478 ± 28). Heart mtDNAcn was significantly increased in the E15 group (control: 413 ± 14; E8: 683 ± 140; E15: 521 ± 32). By contrast, no statistically detectable difference in liver mtDNAcn was observed among the groups (control: 271 ± 40; E8: 325 ± 75; E15: 400 ± 113).

**Figure 5:**
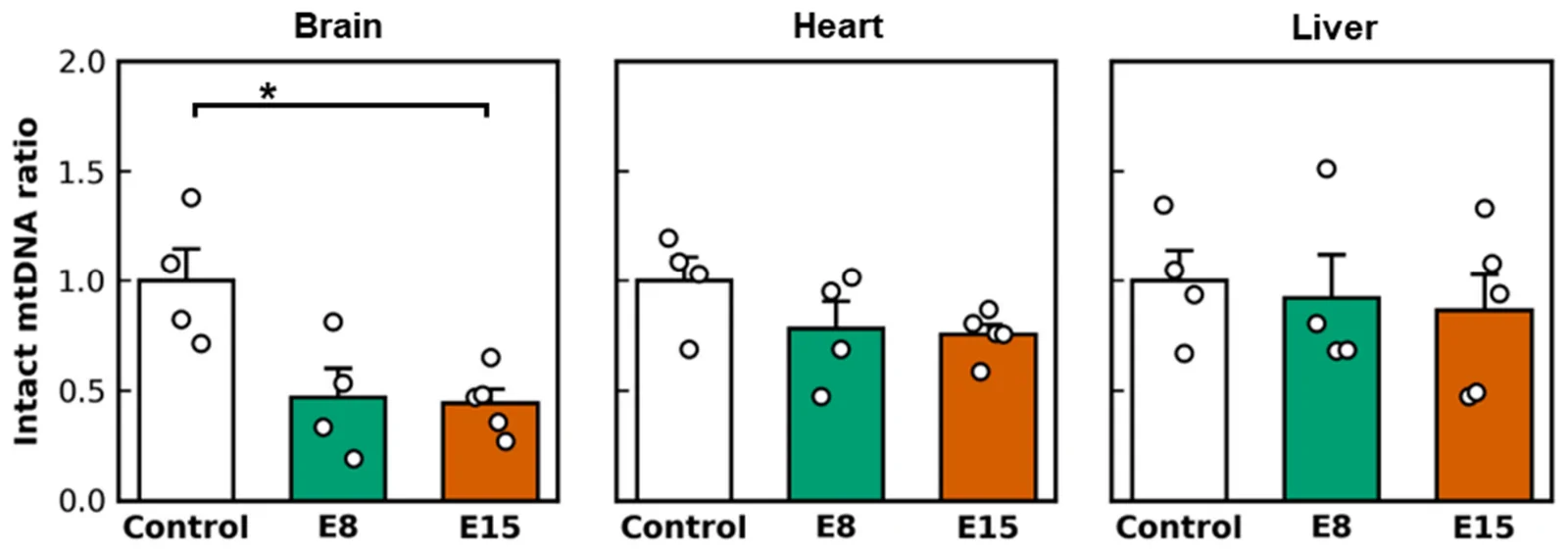
Tissue intact mtDNA ratio in offspring at birth. Data are presented as mean ± SEM; each point represents one litter (Control, *n* = 4; E8, *n* = 4; E15, *n* = 5). Statistical analysis was performed using the Kruskal–Wallis test followed by Steel–Dwass multiple-comparison tests. * *p* < 0.05.

A distinct pattern was observed for the intact mtDNA ratio (Figure 5). The brain intact mtDNA ratio was significantly reduced in the E15 group compared with the control group (control: 1.00 ± 0.15; E8: 0.47 ± 0.14; E15: 0.44 ± 0.06). No statistically detectable group differences in the intact mtDNA ratio were observed in the heart (control: 1.00 ± 0.11; E8: 0.78 ± 0.12; E15: 0.75 ± 0.05) or liver (control: 1.00 ± 0.14; E8: 0.92 ± 0.20; E15: 0.86 ± 0.17).

Because the intact mtDNA ratio is mathematically dependent on mtDNAcn, we additionally examined the 8.2-kb long-fragment PCR-derived quantity before normalization to mtDNAcn (Q). Four-pup values were averaged within each litter, and litter means were used as the experimental units. In the brain, the mean ± SEM values were 0.0227 ± 0.0036, 0.0240 ± 0.0050, and 0.0276 ± 0.0042 (ng/reaction) for the control, E8, and E15 groups, respectively (*p* = 0.589). In the heart, the corresponding values were 0.0246 ± 0.0028, 0.0273 ± 0.0028, and 0.0239 ± 0.0026 (ng/reaction) (*p* = 0.548). In the liver, the values were 0.0251 ± 0.0016, 0.0260 ± 0.0032, and 0.0268 ± 0.0023 (ng/reaction) (*p* = 0.942). Thus, no statistically detectable group difference in the unnormalized 8.2-kb quantity was observed in any organ. The pup-level and litter-level 8.2-kb long-fragment PCR-derived quantity before normalization to mtDNAcn are provided in Supplementary Tables S1 and S2, respectively, and the group-level summary and Kruskal–Wallis test results are provided in Supplementary Table S3.

Linear mixed-effects model results are summarized in Table 1. For mtDNAcn, the exposure group × organ interaction yielded χ^2^ = 24.32 (df = 4, *p* < 0.001), indicating that the pattern of group differences varied among the brain, heart, and liver. The test of whether the E8-versus-E15 contrast varied among organs yielded χ^2^ = 7.77 (df = 2, *p* = 0.021). However, direct comparisons between E8 and E15 within individual organs were not statistically significant after Holm adjustment (brain, *p* = 0.524; heart, *p* = 0.260; liver, *p* = 0.643). Thus, the significant omnibus test indicates that the E8- versus-E15 contrast was not uniform across organs but does not identify a statistically significant E8-versus-E15 difference within any particular organ.

For the intact mtDNA ratio, the exposure group × organ interaction was not statistically significant (χ^2^ = 5.47, df = 4, *p* = 0.243). The test of whether the E8-versus-E15 contrast varied among organs was also not statistically significant (χ^2^ = 0.21, df = 2, *p* = 0.899). Direct comparisons between E8 and E15 within individual organs yielded Holm-adjusted *p* = 1.000 in the brain, heart, and liver (Table 1).

## 3. Discussion

**Figure 6:**
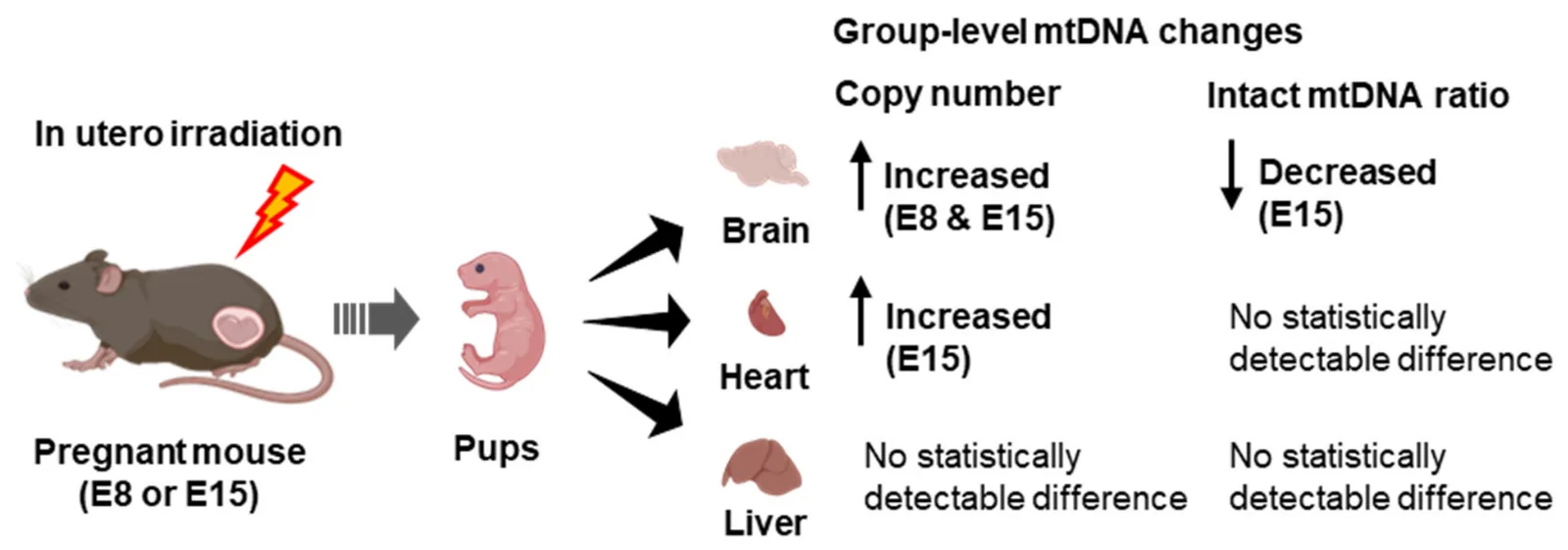
Labels summarize organ-wise comparisons with the control group and do not indicate statistically significant differences between the E8 and E15 exposure groups. Created in BioRender. Fukunaga, H. (2026) https://app.biorender.com/citation/6a635e567a1abfd955d3795b.

The principal finding of this study is that in utero exposure to 0.2 Gy X-rays produced an organ-dependent pattern of mtDNAcn responses in offspring at birth, although no statistically detectable group differences in litter size or neonatal body weight were observed (Figure 6). This finding indicates that gross birth outcomes and molecular endpoints represent different levels of biological observation and that the absence of a detectable difference in conventional birth outcomes does not exclude a molecular response to prenatal irradiation. Within the developmental origins of health and disease (DOHaD) framework [16,17], molecular endpoints such as mtDNAcn may contribute to the characterization of early biological responses to prenatal environmental stress. However, the biological and longer-term significance of these neonatal molecular responses remains unknown and requires further functional and longitudinal investigation.

The observed patterns differed among organs. In the brain, mtDNAcn was higher than in the control group following both E8 and E15 exposure, whereas the intact mtDNA ratio was lower than in the control group following E15 exposure. In the heart, mtDNAcn was higher than in the control group following E15 exposure, whereas no statistically detectable difference in the intact mtDNA ratio was observed. In the liver, no statistically detectable differences in mtDNAcn or the intact mtDNA ratio were observed among the groups. These indices represent related aspects of mitochondrial genomic responses; however, the intact mtDNA ratio is mathematically dependent on mtDNAcn because mtDNAcn is included in its denominator. Therefore, changes in the intact mtDNA ratio cannot be interpreted independently of concurrent changes in mtDNAcn.

Previous studies have reported concurrent increases in mtDNAcn and decreases in long-fragment PCR-based indices following radiation exposure [4,5]. In the present study, however, analysis of the 8.2-kb long-fragment PCR-derived quantity before normalization to mtDNAcn (Q) did not provide evidence of group differences in the brain (*p* = 0.589), heart (*p* = 0.548), or liver (*p* = 0.942). Thus, the lower intact mtDNA ratio observed in the brain following E15 exposure was not accompanied by a statistically detectable group difference in Q. Rather, the lower ratio indicates that the 8.2-kb long-fragment PCR-derived quantity did not increase proportionately with mtDNAcn. Because the intact mtDNA ratio is intended to express the long-fragment PCR-derived quantity relative to total mtDNAcn, this relative decrease remains a feature of the observed mitochondrial genomic response, even in the absence of an absolute reduction in *Q*. Nevertheless, because mtDNAcn is included in the denominator, the ratio is mathematically influenced by concurrent changes in mtDNAcn and must be interpreted together with mtDNAcn. It should not, by itself, be regarded as direct evidence of increased damage to the entire mitochondrial genome or of mitochondrial functional impairment. Further studies using complementary approaches that assess mtDNA damage or amplifiability independently of mtDNAcn will be required to determine the biological basis and functional significance of this finding.

The mixed-effects analysis demonstrated that the pattern of mtDNAcn differences among exposure groups varied across organs (*p* < 0.001). The E8-versus-E15 contrast was also not uniform across organs (*p* = 0.021). However, direct comparisons between E8 and E15 within individual organs were not statistically significant after Holm adjustment (brain, *p* = 0.524; heart, *p* = 0.260; liver, *p* = 0.643). These findings support an organ-dependent pattern of mtDNAcn responses but do not identify a critical gestational window within any individual organ. For the intact mtDNA ratio, neither the exposure group × organ interaction (*p* = 0.243) nor the test of whether the E8-versus-E15 contrast varied among organs (*p* = 0.899) was statistically significant. Therefore, the lower ratio identified in the brain after E15 exposure in the organ-wise analysis should not be interpreted as evidence of either a late-gestation-specific response or brain-specific vulnerability.

The present findings should not be interpreted as evidence of mitochondrial dysfunction, developmental impairment, or subsequent disease risk. Mitochondrial function, histopathological alterations, oxidative stress, and postnatal outcomes were not assessed in this study. Moreover, the exposure conditions and developmental characteristics of the mouse model preclude direct extrapolation to human pregnancy or estimation of human health risks. The mtDNA parameters examined here should therefore be regarded as molecular endpoints for characterizing neonatal responses to prenatal irradiation, rather than as established biomarkers of adverse health effects.

From a translational perspective, our findings suggest that organ-specific mitochondrial susceptibility may underlie differential responses to early-life environmental stress and may represent early events within the DOHaD framework. However, the present findings do not establish the clinical or prognostic validity of mtDNAcn or long-fragment mtDNA amplifiability as biomarkers. The biological significance of these indices following irradiation may depend on developmental context, exposure timing, and tissue-specific mitochondrial genome maintenance. However, these findings should not be interpreted as evidence of adverse developmental or long-term health effects, as such outcomes were not evaluated in the present study. The present study does not provide a risk estimate for human pregnancy. Rather, the findings suggest that mitochondrial genome quantity and the amplifiability of a defined long mtDNA region may complement conventional developmental outcomes in environmental health research, particularly within the DOHaD framework.

Several limitations should be acknowledged. First, this study was designed to resolve mitochondrial genomic endpoints rather than mitochondrial function; therefore, respiratory capacity, reactive oxygen species production, antioxidant responses, and DNA repair signaling were not directly assessed. Second, the molecular mechanisms underlying the observed organ-dependent mitochondrial genomic responses remain unclear. Third, the study was limited to three neonatal organs and one postnatal time point, and whether the observed mitochondrial responses persist, recover, or evolve after birth remains unknown. Fourth, although litter means were used as the experimental units, the number of independent litters was limited (*n* = 4, 4, and 5 in the control, E8, and E15 groups, respectively). No a priori sample size calculation was performed, and no primary outcome was prespecified. Consequently, the statistical estimates are subject to substantial uncertainty, particularly for interaction tests and organ-specific comparisons, and non-significant findings should not be interpreted as evidence for the absence of an effect. The present findings should therefore be regarded as exploratory and require confirmation in studies involving larger numbers of independent litters. Future studies incorporating larger numbers of independent litters, longitudinal sampling, and functional mitochondrial assays will be important for confirming the durability and biological consequences of these responses. In addition, the sex of the offspring was not determined in this study, and an unequal distribution of males and females among the four pups selected from each litter may have contributed to variability in the observed responses or to differences between groups. Future studies should determine offspring sex and incorporate sex-balanced or sex-stratified analyses.

## 4. Materials and Methods

### 4.1. Study Design

As shown in Figure 1, pregnant dams were assigned prospectively but non-randomly to the three experimental groups according to the predetermined experimental schedule: control (sham-irradiated, *n* = 4), E8-irradiated (*n* = 4), and E15-irradiated (*n* = 5). No random allocation procedure was used. The pregnant dam and the resulting litter constituted the experimental unit for gestational exposure.

Irradiated dams received a single whole-body dose of 0.2 Gy X-rays at E8 or E15, which were used to represent early- and late-gestation exposure windows, respectively. The dose was selected based on our previous study in which in utero exposure to 0.2 Gy X-rays did not produce statistically detectable alterations in litter size or neonatal body weight [5].

At E8, before irradiation in each group, maternal body weight was recorded and peripheral blood was collected for mtDNAcn analysis. On the day of birth, litter size and neonatal body weight were recorded. Newborn pups were then euthanized by an overdose of inhaled isoflurane before tissue collection. The brain, heart, and liver were selected because mitochondrial respiratory-chain composition, oxidative capacity, and energy metabolism undergo substantial early postnatal maturation in these organs [13,14,15].

Four pups per litter were randomly selected for mtDNAcn and long-fragment PCR-based intact mtDNA ratio measurements. This random selection represented within-litter subsampling and did not constitute randomization of the experimental units. The four pup-level values were averaged within each litter, and the litter mean was used as the experimental unit for statistical analysis. The sex of the newborn pups was not determined, and pup selection was therefore not stratified by sex. Consequently, sex-specific effects could not be assessed.

All animals were housed under identical environmental conditions, and experimental procedures were conducted using the same protocol for all groups to minimize potential confounding factors. This study is reported in accordance with the ARRIVE 2.0 guidelines. Blinding was not performed during sample collection, laboratory analyses, or data analysis.

The animals, litters, biological samples, and data used in the present study were entirely independent of those used in our previous study [5]. Whereas our previous study examined blood-derived mtDNA parameters following E8 exposure at two weeks of age, the present study evaluated the brain, heart, and liver at birth following exposure at E8 or E15.

### 4.2. Animals

Pregnant C57BL/6N mice (11–13 weeks of age) were obtained from CLEA Japan (Tokyo, Japan). The animals were housed individually under controlled conditions at 23 ± 2 °C and 50 ± 10% relative humidity, with a 12-h light/dark cycle and free access to food and water. The cages contained paper-based bedding, and a cardboard shelter was provided as environmental enrichment. Following arrival at the animal facility, the mice were allowed to acclimatize for 1 day before blood collection and irradiation. Animal welfare was monitored daily by visual inspection without disturbing the cages, including assessment of general condition, activity, appearance, feeding and drinking behavior, and abnormal behavior.

### 4.3. Irradiation Settings

A single whole-body exposure of 0.2 Gy X-rays was delivered to pregnant mice using an MBR-1520R-4 X-ray irradiator (Hitachi Power Solutions, Hitachi-shi, Ibaraki, Japan) operated at 150 kVp and 20 mA with a 1.0-mm aluminum filter. The source-to-animal distance was 550 mm. The dose rate at the animal position was approximately 1.80 Gy/min.

The air kerma at the animal position was measured using a semiflex ionization chamber (type 31010; PTW Freiburg GmbH, Freiburg, Germany). The dosimeter was subsequently moved to a position that did not interfere with the experimental setup, and the air kerma was measured again. The irradiation condition at the animal position was determined based on the relative measurements obtained at the two positions.

During irradiation, mice were placed in a circular mouse holder, with the center of the maternal trunk positioned at the irradiation center. The animals were aligned along the longitudinal and lateral axes. Field uniformity was not independently evaluated during the present experiment. The absorbed dose to the embryos or fetuses was not directly measured or estimated; therefore, the reported 0.2 Gy represents the irradiation condition based on air-kerma measurements at the maternal animal position and should not be interpreted as a directly determined fetal absorbed dose.

### 4.4. DNA Extraction

Genomic DNA was isolated from 50–100 μL of maternal peripheral blood using the ISOSPIN Blood & Plasma DNA Kit (Nippon Gene, Tokyo, Japan) in accordance with the manufacturer’s protocol. Total DNA from offspring tissues was extracted using the NucleoSpin Tissue Kit (Macherey-Nagel, Düren, Germany) following the supplier’s instructions. All samples were stored at −20 °C until analysis.

### 4.5. Estimation of Mitochondrial DNA Copy Number and Intact Ratio

DNA concentration and purity were measured spectrophotometrically, and samples were adjusted to the DNA concentrations specified below for each assay.

To measure mtDNAcn relative to nuclear DNA copy number in mice, we used a real-time qPCR assay (Mouse Feeder Cell Quantification Kit, RR290; Takara Bio Inc., Kusatsu, Shiga, Japan). The assay targets the mitochondrial *COXII* region and the nuclear *Rplp1* gene. The specific primer sequences and amplicon lengths are proprietary and are not disclosed by the manufacturer. Real-time PCR was performed using an Applied Biosystems StepOne Real-Time PCR Analysis System (Thermo Fisher Scientific Inc., Waltham, MA, USA). According to the manufacturer’s protocol, each 25-μL reaction contained 12.5 μL of TB Green Premix Ex Taq II (Tli RNaseH Plus, Takara-Bio Inc., Kusatsu, Shiga, Japan), 1.0 μL of the corresponding primer mix, 0.5 μL of ROX Reference Dye, 9.0 μL of distilled water, and 2.0 μL of DNA template at 5 ng/μL, corresponding to 10 ng of DNA per reaction. Thermal cycling consisted of an initial denaturation at 95 °C for 30 s, followed by 40 cycles of 95 °C for 5 s and 60 °C for 30 s, followed by melting-curve analysis. Relative mtDNAcn was calculated based on the difference in *Ct* values between mitochondrial and nuclear genes (Δ*Ct* method) [18]. Each sample was analyzed once without technical replication; therefore, intra-assay variability was not estimated. No detectable amplification was observed in the negative controls, and *Ct* values were not assigned by the PCR instrument because the fluorescence signals remained below the detection threshold.

Assessment of the amplifiability of a defined long mtDNA region was performed using a long-fragment PCR-based approach (Mouse Mitochondrial DNA Damage Analysis Kit, DD2M; Detroit R&D, Detroit, MI, USA). In this method, an 8.2-kb region of mtDNA is amplified. Efficient amplification requires uninterrupted polymerase progression across the defined target region; therefore, DNA strand breaks and polymerase-blocking lesions located within this region can reduce the amount of amplification product. Accordingly, this assay evaluates the amplifiability of the defined 8.2-kb region and does not directly assess the integrity of the entire mitochondrial genome or identify specific types of mtDNA lesions. The specific primer sequences and genomic coordinates of this proprietary assay are not provided by the manufacturer. The long-fragment amplification was performed in a total reaction volume of 20 μL containing 10.0 μL of 2× QPCR concentrated buffer, 4.0 μL of 5× Enhancer, 5.0 μL of QPCR primer mix (2 μM each forward and reverse primer), and 1.0 μL of DNA template at 50 ng/μL, corresponding to 50 ng of DNA per reaction. Amplification was performed using the Applied Biosystems StepOne Real-Time PCR Analysis System. Thermal cycling consisted of 98 °C for 30 s, followed by 30 cycles of 98 °C for 10 s, 67 °C for 10 s, and 72 °C for 4 min, with a final extension at 72 °C for 10 min and subsequent holding at 4 °C.

The resulting long-fragment PCR products were diluted 10-fold with nuclease-free water before real-time PCR quantification. Each 20-μL real-time PCR reaction contained 10.0 μL of SYBR Green mix (PowerTrack™ SYBR Green Master Mix for qPCR, A46109; Thermo Fisher Scientific Inc., Waltham, MA, USA), 7.1 μL of nuclease-free water, 0.9 μL of real-time primer mix (5 μM each forward and reverse primer), and 2.0 μL of diluted PCR product. Thermal cycling consisted of 50 °C for 2 min and 95 °C for 10 min, followed by 40 cycles of 95 °C for 15 s and 60 °C for 60 s. Each sample was analyzed once without technical replication; therefore, intra-assay variability was not estimated. Negative controls were included in the real-time PCR analysis. No detectable amplification was observed in the negative controls, and *Ct* values were not assigned by the PCR instrument because the fluorescence signals remained below the detection threshold.

A standard curve was generated using serial dilutions of the 8.2-kb DNA standard at 2 ng, 200 pg, 20 pg, 2 pg, 0.2 pg, and 0.02 pg per reaction. The 8.2-kb long-fragment PCR-derived quantity before normalization to mtDNAcn for each sample was calculated from the *Ct* value according to the standard curve:

(1) $$Q_{i}=10^{a\;Ct_{i}+b}$$

where $Q_{i}$ is the 8.2-kb long-fragment PCR-derived quantity (ng/reaction) before normalization to mtDNAcn for sample i, $Ct_{i}$ is the Ct value of sample i, and a and b are the slope and intercept, respectively, obtained from the standard curve ($a=-0.2794,\;b=3.4459;R^{2}=0.989$). The amplification efficiency of the real-time qPCR used to quantify the diluted 8.2-kb long-fragment PCR products was 90.3%.

For each sample, $Q_{i}$ was then normalized to the mtDNA copy number of the same sample:

(2) $$R_{i}=\frac{Q_{i}}{{mtDNAcn}_{i}}$$

where ${mtDNAcn}_{i}$ represents the mtDNAcn of sample i.

The final intact mtDNA ratio was then calculated by normalizing $R_{i}$ to the mean R value of the corresponding organ-specific control group:

(3) $${Intact\;mtDNA\;ratio}_{i}=\frac{R_{i}}{\frac{1}{n_{C,j}}\sum_{k\in C,j}R_{k}}.$$

where ${Intact\;mtDNA\;ratio}_{i}$ represents the intact mtDNA ratio for sample i, *C*, *j* denotes the control samples for the corresponding organ j, and $n_{C,j}$ is the number of control samples for that organ. Consequently, the mean intact mtDNA ratio of the control group is 1.0 for each organ.

### 4.6. Statistical Analysis

Normality was assessed using the Shapiro–Wilk test. Data are presented as mean ± SEM.

Maternal variables and litter size were analyzed using one-way analysis of variance (ANOVA) followed by Tukey–Kramer multiple-comparison tests. For neonatal body weight, tissue mtDNAcn, and intact mtDNA ratios, measurements from four pups were averaged within each litter before analysis. Neonatal body weight was analyzed using one-way ANOVA followed by Tukey–Kramer multiple-comparison tests. Tissue mtDNAcn and intact mtDNA ratios were analyzed using the Kruskal–Wallis test followed by Steel–Dwass multiple-comparison tests. Statistical significance was set at *p* < 0.05.

To evaluate whether exposure-related responses differed among organs while accounting for the dependence among measurements obtained from the same litter, an additional linear mixed-effects model was applied to the litter-level data. Litter-level mtDNAcn and intact mtDNA ratio values were natural-log transformed before mixed-model analysis.

The model was specified as

(4) $$\log Y_{ij}=\beta_{0}+\beta_{Group}+\beta_{Organ}+\beta_{Group\times Organ}+b_{i}+\varepsilon_{ij}$$

where $Y_{ij}$ represents the litter-level outcome for litter i and organ j, $b_{i}$ is the litter-specific random intercept, and $\varepsilon_{ij}$ is the residual error. The random intercept was assumed to follow $b_{i}\sim N(0,\tau^{2})$. To accommodate differences in variability among organs, separate residual variances were estimated for the brain, heart, and liver, such that $\varepsilon_{ij}\sim N(0,{\sigma_{j}}^{2})$. Models were fitted using maximum likelihood.

The overall exposure group × organ interaction was evaluated by comparing the full model containing the interaction with an additive model without the group × organ interaction using a 4-df likelihood-ratio test. To specifically determine whether the E8-versus-E15 difference varied among organs, the full model was compared with a constrained model in which E8 and E15 shared the same organ-interaction effects, using a 2-df likelihood-ratio test. E8 and E15 were also directly compared within each organ using model-based Wald contrasts on the logarithmic scale. Two-sided *p* values were calculated using the standard normal distribution. Contrast estimates and 95% confidence intervals were back-transformed and are reported as E8/E15 ratios. *p* values for the three organ-specific E8-versus-E15 comparisons were adjusted using the Holm method.

All statistical analyses were performed using Python 3.13.9 with NumPy 2.3.5, pandas 2.3.3, and SciPy 1.16.3.

## 5. Conclusions

In utero exposure to 0.2 Gy X-rays produced an organ-dependent pattern of mtDNAcn responses in neonatal mice without statistically detectable group differences in litter size or neonatal body weight. Organ-wise comparisons showed higher brain mtDNAcn following E8 and E15 exposure and higher heart mtDNAcn following E15 exposure; however, direct comparisons between E8 and E15 were not statistically significant within any organ. Although the intact mtDNA ratio was lower in the brain following E15 exposure in the organ-wise analysis, mixed-effects analysis did not support organ- or gestational-stage-dependent patterns in this measure. These findings characterize neonatal mitochondrial genomic responses under the present experimental conditions but do not establish mitochondrial dysfunction, adverse developmental effects, or longer-term health consequences. Given the limited number of independent litters, the findings should be regarded as exploratory. Larger studies incorporating functional mitochondrial assays and postnatal follow-up are needed to determine the biological significance and persistence of these responses.

## Figures and Tables

**Table 1:** Linear mixed-effects model analyses of mtDNAcn and intact mtDNA ratio. Estimates for organ-specific comparisons are back-transformed E8/E15 ratios. Interaction *p* values were obtained using likelihood-ratio tests. Organ-specific comparisons between the E8 and E15 exposure groups were performed using two-sided Wald tests, and *p* values were adjusted using the Holm method.

Outcome	Comparison/Test	Organ	Estimate	95% CI	*p*-Value
mtDNAcn	Exposure group × organ interaction	—	—	—	<0.001
	E8-versus-E15 × organ interaction	—	—	—	0.021
	E8-to-E15 comparison	Brain	0.87	0.69–1.11	0.524
		Heart	1.25	0.97–1.61	0.260
		Liver	0.87	0.49–1.55	0.643
intact mtDNA ratio	Exposure group × organ interaction	—	—	—	0.243
	E8-versus-E15 × organ interaction	—	—	—	0.899
	E8-to-E15 comparison	Brain	0.96	0.56–1.63	1.000
		Heart	1.00	0.75–1.34	1.000
		Liver	1.09	0.71–1.67	1.000

## Data Availability

The original contributions presented in this study are included in the article/Supplementary Materials. Further inquiries can be directed to the corresponding author.

## Supplementary Materials

The following supporting information can be downloaded at: https://www.mdpi.com/article/10.3390/ijms27177671/s1.

## References

[1] Kam W.W.Y., Banati R.B. (2013). Effects of Ionizing Radiation on Mitochondria. Free Radic. Biol. Med..

[2] Zhou X., Li N., Wang Y., Wang Y., Zhang X., Zhang H. (2011). Effects of X-Irradiation on Mitochondrial DNA Damage and Its Supercoiling Formation Change. Mitochondrion.

[3] Zhang H., Maguire D., Swarts S., Sun W., Yang S., Wang W., Liu C., Zhang M., Zhang D., Zhang L. (2009). Replication of Murine Mitochondrial DNA Following Irradiation. Adv. Exp. Med. Biol..

[4] Seino R., Takahashi Y., Fukunaga H. (2025). Radiation-Induced Changes in Peripheral Blood-Derived Mitochondrial DNA Copy Number and Heteroplasmy in Mice: Potential Biomarkers of Radiation Exposure. Biomed. Res..

[5] Seino R., Kubo H., Ikeda A., Fukunaga H. (2026). Mitochondrial DNA Alterations in Mothers and Offspring Following in Utero Exposure to Ionizing Radiation. Free Radic. Biol. Med..

[6] Cao J., Spielmann M., Qiu X., Huang X., Ibrahim D.M., Hill A.J., Zhang F., Mundlos S., Christiansen L., Steemers F.J. (2019). The Single-Cell Transcriptional Landscape of Mammalian Organogenesis. Nature.

[7] Xie P., Shen J., Yang Y., Wang X., Liu W., Cao H., Zheng Y., Wu C., Mao G., Chen L. (2025). Digital Reconstruction of Full Embryos during Early Mouse Organogenesis. Cell.

[8] Sreetharan S., Thome C., Tharmalingam S., Jones D.E., Kulesza A.V., Khaper N., Lees S.J., Wilson J.Y., Boreham D.R., Tai T.C. (2017). Ionizing Radiation Exposure During Pregnancy: Effects on Postnatal Development and Life. Radiat. Res..

[9] ICRP (2007). The 2007 Recommendations of the International Commission on Radiological Protection. ICRP Publication 103. Ann. ICRP.

[10] Streffer C., Shore R., Konermann G., Meadows A., Uma Devi P., Preston Withers J., Holm L.E., Stather J., Mabuchi K., Withers H.R. (2003). Biological Effects after Prenatal Irradiation (Embryo and Fetus). A Report of the International Commission on Radiological Protection. Ann. ICRP.

[11] Grison S., Braga-Tanaka I.I.I.I., Baatout S., Klokov D. (2024). In Utero Exposure to Ionizing Radiation and Metabolic Regulation: Perspectives for Future Multi- and Trans-Generation Effects Studies. Int. J. Radiat. Biol..

[12] Kelly R.D.W., Mahmud A., McKenzie M., Trounce I.A., St John J.C. (2012). Mitochondrial DNA Copy Number Is Regulated in a Tissue Specific Manner by DNA Methylation of the Nuclear-Encoded DNA Polymerase Gamma A. Nucleic Acids Res..

[13] Siragusa M., Yoval-Sánchez B., Guerrero I., Galkin A. (2025). Postnatal Developmental Dynamics of Mitochondrial Complex I in Mouse Tissues. Am. J. Physiol. Cell Physiol..

[14] Piquereau J., Novotova M., Fortin D., Garnier A., Ventura-Clapier R., Veksler V., Joubert F. (2010). Postnatal Development of Mouse Heart: Formation of Energetic Microdomains. J. Physiol..

[15] Valcarce C., Navarrete R.M., Encabo P., Loeches E., Satrústegui J., Cuezva J.M. (1988). Postnatal Development of Rat Liver Mitochondrial Functions: The Roles of Protein Synthesis and of Adenine Nucleotides. J. Biol. Chem..

[16] Gluckman P.D., Hanson M.A. (2004). Living with the Past: Evolution, Development, and Patterns of Disease. Science.

[17] Hanson M.A., Gluckman P.D. (2014). Early Developmental Conditioning of Later Health and Disease: Physiology or Pathophysiology?. Physiol. Rev..

[18] Rooney J.P., Ryde I.T., Sanders L.H., Howlett E.H., Colton M.D., Germ K.E., Mayer G.D., Greenamyre J.T., Meyer J.N. (2015). PCR Based Determination of Mitochondrial DNA Copy Number in Multiple Species. Methods Mol. Biol..

