# Supplementary material for: Organ-Specific Mitochondrial DNA Copy Number Responses in Neonatal Mice Following in Utero Irradiation
Source: Int J Mol Sci. 2026 Aug 27;27(17):7671. doi: 10.3390/ijms27177671 (PMC13565987; doi:10.3390/ijms27177671)
Supplement: Supplementary file 1 [file ijms-27-07671-s001.zip › ijms-4486200-supplementary.pdf]

**Table S1. Pup-level 8.2-kb long-fragment PCR-derived quantities before normalization to mtDNA copy number in neonatal mouse organs.**

The values shown represent the 8.2-kb long-fragment PCR-derived quantities calculated from the standard curve before normalization to mtDNA copy number. Four pups were analyzed per litter. Pup-level values were averaged within each litter to obtain the litter-level means used as the independent experimental units for statistical analysis.

| Group | Mother ID | Pup ID | Brain (ng/reaction) | Heart (ng/reaction) | Liver (ng/reaction) |
|---|---|---|---|---|---|
| Control | Control -1 | Pup 1 | 0.0180 | 0.0320 | 0.0198 |
| Control | Control -1 | Pup 2 | 0.0463 | 0.0099 | 0.0298 |
| Control | Control -1 | Pup 3 | 0.0301 | 0.0248 | 0.0093 |
| Control | Control -1 | Pup 4 | 0.0159 | 0.0521 | 0.0424 |
| Control | Control -2 | Pup 1 | 0.0314 | 0.0085 | 0.0141 |
| Control | Control -2 | Pup 2 | 0.0182 | 0.0399 | 0.0112 |
| Control | Control -2 | Pup 3 | 0.0116 | 0.0138 | 0.0329 |
| Control | Control -2 | Pup 4 | 0.0219 | 0.0343 | 0.0280 |
| Control | Control -3 | Pup 1 | 0.0421 | 0.0124 | 0.0229 |
| Control | Control -3 | Pup 2 | 0.0120 | 0.0199 | 0.0085 |
| Control | Control -3 | Pup 3 | 0.0331 | 0.0278 | 0.0485 |
| Control | Control -3 | Pup 4 | 0.0290 | 0.0080 | 0.0168 |
| Control | Control -4 | Pup 1 | 0.0179 | 0.0577 | 0.0505 |
| Control | Control -4 | Pup 2 | 0.0081 | 0.0172 | 0.0128 |
| Control | Control -4 | Pup 3 | 0.0183 | 0.0241 | 0.0206 |
| Control | Control -4 | Pup 4 | 0.0095 | 0.0114 | 0.0339 |
| E8 | E8 -1 | Pup 1 | 0.0123 | 0.0255 | 0.0078 |
| E8 | E8 -2 | Pup 1 | 0.0357 | 0.0157 | 0.0103 |
| E8 | E8 -3 | Pup 1 | 0.0090 | 0.0227 | 0.0092 |
| E8 | E8 -4 | Pup 1 | 0.0128 | 0.0363 | 0.0131 |
| E8 | E8 -1 | Pup 2 | 0.0538 | 0.0346 | 0.0520 |
| E8 | E8 -2 | Pup 2 | 0.0568 | 0.0371 | 0.0258 |
| E8 | E8 -3 | Pup 2 | 0.0138 | 0.0077 | 0.0183 |
| E8 | E8 -4 | Pup 2 | 0.0132 | 0.0102 | 0.0145 |
| E8 | E8 -1 | Pup 3 | 0.0075 | 0.0089 | 0.0157 |
| E8 | E8 -2 | Pup 3 | 0.0212 | 0.0134 | 0.0288 |
| E8 | E8 -3 | Pup 3 | 0.0490 | 0.0313 | 0.0447 |
| E8 | E8 -4 | Pup 3 | 0.0096 | 0.0415 | 0.0363 |
| E8 | E8 -1 | Pup 4 | 0.0351 | 0.0548 | 0.0402 |
| E8 | E8 -2 | Pup 4 | 0.0245 | 0.0630 | 0.0314 |
| E8 | E8 -3 | Pup 4 | 0.0212 | 0.0186 | 0.0604 |
| E8 | E8 -4 | Pup 4 | 0.0078 | 0.0147 | 0.0082 |
| E15 | E15 -1 | Pup 1 | 0.0110 | 0.0667 | 0.0240 |
| E15 | E15 -2 | Pup 1 | 0.0414 | 0.0094 | 0.0162 |
| E15 | E15 -3 | Pup 1 | 0.0199 | 0.0084 | 0.0214 |
| E15 | E15 -4 | Pup 1 | 0.0415 | 0.0128 | 0.0175 |
| E15 | E15 -5 | Pup 1 | 0.0083 | 0.0358 | 0.0192 |
| E15 | E15 -1 | Pup 2 | 0.0289 | 0.0119 | 0.0647 |
| E15 | E15 -2 | Pup 2 | 0.0155 | 0.0165 | 0.0583 |
| E15 | E15 -3 | Pup 2 | 0.0667 | 0.0490 | 0.0322 |
| E15 | E15 -4 | Pup 2 | 0.0126 | 0.0385 | 0.0252 |
| E15 | E15 -5 | Pup 2 | 0.0328 | 0.0267 | 0.0293 |
| E15 | E15 -1 | Pup 3 | 0.0604 | 0.0289 | 0.0272 |
| E15 | E15 -2 | Pup 3 | 0.0092 | 0.0336 | 0.0434 |
| E15 | E15 -3 | Pup 3 | 0.0374 | 0.0237 | 0.0276 |
| E15 | E15 -4 | Pup 3 | 0.0127 | 0.0164 | 0.0121 |
| E15 | E15 -5 | Pup 3 | 0.0167 | 0.0177 | 0.0087 |
| E15 | E15 -1 | Pup 4 | 0.0424 | 0.0214 | 0.0116 |
| E15 | E15 -2 | Pup 4 | 0.0522 | 0.0140 | 0.0074 |
| E15 | E15 -3 | Pup 4 | 0.0239 | 0.0280 | 0.0097 |
| E15 | E15 -4 | Pup 4 | 0.0092 | 0.0110 | 0.0563 |
| E15 | E15 -5 | Pup 4 | 0.0089 | 0.0074 | 0.0247 |

**Table S2. Litter-level mean 8.2-kb long-fragment PCR-derived quantities before normalization to mtDNA copy number in neonatal mouse organs.**

Values represent litter-level means calculated by averaging measurements from four pups within each litter. The litter was treated as the independent experimental unit for statistical analysis (Control, n = 4 litters; E8, n = 4 litters; E15, n = 5 litters).

| Group | Mother ID | Brain (ng/reaction) | Heart (ng/reaction) | Liver (ng/reaction) |
|---|---|---|---|---|
| Control | Control -1 | 0.0276 | 0.0297 | 0.0253 |
| Control | Control -2 | 0.0208 | 0.0241 | 0.0215 |
| Control | Control -3 | 0.0290 | 0.0170 | 0.0242 |
| Control | Control -4 | 0.0135 | 0.0276 | 0.0295 |
| E8 | E8 -1 | 0.0272 | 0.0310 | 0.0289 |
| E8 | E8 -2 | 0.0345 | 0.0323 | 0.0241 |
| E8 | E8 -3 | 0.0233 | 0.0201 | 0.0331 |
| E8 | E8 -4 | 0.0108 | 0.0257 | 0.0180 |
| E15 | E15 -1 | 0.0357 | 0.0322 | 0.0319 |
| E15 | E15 -2 | 0.0296 | 0.0184 | 0.0313 |
| E15 | E15 -3 | 0.0370 | 0.0273 | 0.0227 |
| E15 | E15 -4 | 0.0190 | 0.0197 | 0.0278 |
| E15 | E15 -5 | 0.0167 | 0.0219 | 0.0205 |

**Table S3. Group-level summary and statistical analysis of 8.2-kb long-fragment PCR-derived quantities before normalization to mtDNA copy number.**

Values are presented as mean ± SEM of litter-level means (Control, n = 4 litters; E8, n = 4 litters; E15, n = 5 litters). Group differences within each organ were evaluated using the Kruskal–Wallis test. Post hoc pairwise comparisons were not performed because the overall test yielded $p > 0.05$ for all organs.

| Organ | Control mean ± SEM (ng/reaction) | E8 mean ± SEM (ng/reaction) | E15 mean ± SEM (ng/reaction) | *p*-value |
|---|---|---|---|---|
| Brain | 0.0227 ± 0.0036 | 0.0240 ± 0.0050 | 0.0276 ± 0.0042 | 0.589 |
| Heart | 0.0246 ± 0.0028 | 0.0273 ± 0.0028 | 0.0239 ± 0.0026 | 0.548 |
| Liver | 0.0251 ± 0.0016 | 0.0260 ± 0.0032 | 0.0268 ± 0.0023 | 0.942 |
